# Criminal Minds: Neuromodulation of the Psychopathic Brain

**DOI:** 10.3389/fnhum.2014.00124

**Published:** 2014-03-05

**Authors:** Sergio Canavero

**Affiliations:** ^1^Turin Advanced Neuromodulation Group, Turin, Italy

**Keywords:** criminal mind, cortical stimulation, neuroimaging, psychosurgery, ethics, medical, psychopathy

Although the definition of criminal behavior is fraught with controversy, with single acts “criminalized” or “decriminalized” according to time and place, and as such being observed in individuals of all sorts, there seems to be an agreement across the board that the truly dangerous subjects are psychopaths and the subjects affected by the Antisocial Personality Disorder (Janowsky, 2008), often repeat offenders. Psychopaths exhibit callousness, lack of empathy or emotional depth, and lack of genuine remorse for their antisocial actions. Although distinct in many regards, a subset of paraphilic subjects too can become dangerous, for instance those suffering sexual sadism, which may involve killing of the victim.

Axis II personality disorder (APD) is thought to be among the most treatment refractory of DSM APDs. Overall, there is little evidence that drugs are reliably effective, and the same conclusion applies to many psychotherapies (Glenn and Raine, 2013). Prognosis appears to be poor, despite psychological, or drug therapy (Balon and Segraves, 2008). Although hypnosis can manipulate moral judgments (Whetley and Haidt, 2005), this modality has proved too erratic in criminal behavior reconditioning.

The advent of *in vivo* neuroimaging allowed the dissection of brain regions involved in such dysfunctions. A wide range of brain areas has been implicated, including the frontal cortex [dorsolateral prefrontal cortex (DLPFC), ventromedial/orbitofrontal cortex (VMPFC/OFC), and the amygdala]. Structural abnormalities include reduced prefrontal gray matter in APD and psychopaths (e.g., Yang et al., 2005; Baron-Cohen, 2011). Importantly, imaging data point to a difference between APD and psychopathy, in that the former have poor impulse control (DLPFC dysfunction), whereas the latter have lack of empathy (non-underactive DLPFC) (Greene, 2009; Baron-Cohen, 2011).

So-called ablative functional neurosurgery, whereby irreversible brain lesions are achieved by various means, has been employed mostly in the past to treat such patients. Sano and Mayanagi (1988) submitted to stereotactic posterior hypothalamotomy, a series of 60 children with violent, aggressive behavior, and a history of epileptic seizures and mental retardation, with good long-term control in many. Other authors reported similar results in this group of patients (e.g., Ramamurthi, 1988). Stereotactic amygdalotomy too has been applied since 1961 for the treatment of severe aggressive behavior, with improvement varying between 33 and 100%, many in the long term (Mpakopoulou et al., 2008). Dieckmann et al. (1988) reported excellent results for the control of aggressive sexual behavior with hypothalamotomy, although the pedophilic character of such patients was retained (see also Roeder, 1966).

Unfortunately, stereotactic neurosurgery is associated with a mortality rate which, according to the depth of penetration of the surgical probes, is not trivial. Thus, it cannot be offered on a routine basis.

Deep brain stimulation (DBS), a reversible, stimulatory technique whereby electrodes are inserted deep into the brain in a targeted fashion, has emerged as a viable option for the treatment of neurological and psychiatric disorders, including aggressive behavior (Arle and Shils, 2011). A report described limbic DBS for intermittent explosive disorder (Maley et al., 2010) and others exist. Even for drug addiction – which often leads to criminal behavior, both ablative and modulatory neurosurgery have been employed with initial promising results (Stelten et al., 2008). Heath (1964) already experimented with septal DBS in psychiatric subjects in order to engage “pleasure” circuits with an eye to preemptive “mind control” of aberrant behaviors. Unfortunately, DBS too carries a small risk of mortality (roughly 0.4%) and disabling morbidity (roughly 1%) (Arle and Shils, 2011).

It is my contention that a strong case can be made for the experimental application of cortical neuromodulation as applied to the control of psychopaths and repeat offenders. This is a risk free, zero-mortality, and zero-morbidity neuromodulatory technique (Canavero, 2009). It modulates the excitability/activity of cortical and related sub-cortical networks involved in pathophysiological disorders, including those of psychiatric classification.

**PREMISE 1**. The Scottish philosopher Hume (1739) referred to humans as “*slaves of the passions*”, and this engendered “immoral” behavior, including crime. More than two centuries later, Delgado (1969), the great neurologist, wrote: “*Some of our present problems derive from the lack of balance between material and mental evolution. We are civilized in our physical ecological accomplishments but barbaric in our psychological responses. Within some limits we can control atoms, trees, and animals, while we have not learned to control ourselves. New solutions are needed in order to civilize our psyche, consciously to organize our efforts to develop a future psychocivilized society* … *why after several thousand years of civilization are human beings continuing to torture and kill each other?* … *the direction of the colossal forces discovered by man requires the development of mental qualities able to apply intelligence not only to the domination of nature but also to the civilization of the human psyche*”.

His solution was the electrical stimulation of the brain. In a much advertised feat, he stopped a raging bull hurtling toward him by simply activating an electrode implanted in the animal’s caudate nucleus by a radio signal. Similarly, he demonstrated the feasibility of controlling human behavior via radio-controlled implanted stimulatory apparatuses. Unfortunately, these first “mind control” attempts required the insertion of electrodes inside or in contact with the brains, casting a pall on the whole undertaking. The unfolding of the psychosurgery saga in the 1960–1970s accelerated the demise of such developments.

**PREMISE 2**. Free will is a mere illusion: a large body of evidence points to the unescapable conclusion that the pursuit of goals that we consciously set and adopt is prepared unconsciously (Wegner, 2002). Goals themselves can arise and operate unconsciously (Custers and Aarts, 2010), with the brain easily deceived and manipulated by external factors (Fine, 2006), including subliminal stimuli (Custers and Aarts, 2010). This fact is still being downplayed or even ignored by many, as this casts a pall on how we should judge criminal behavior (“*He did it, but is not responsible*”). On the other hand, this opens the way to neurosurgical modulation of behavior (presently pursued for other psychiatric indications: Arle and Shils, 2011) in the criminal subject. The goal is redirecting the action course of the criminal behavior by “rewriting” the original priming signal to commit an antisocial act. This should not come as a surprise. Psychopathic behavior is a purely biological epiphenomenon and can be induced. For instance, Blair and Cipolotti (2000) reported a patient (J.S.) who, following trauma to the right frontal region, including the orbitofrontal cortex, presented with “acquired sociopathy”. His behavior was notably aberrant and marked by high levels of aggression and a callous disregard for others (see also Burns and Swerdlow, 2003). Moral reasoning is most usefully thought of as an attempt to explain the cause and effect of our moral intuitions that draws upon all available explicit information about a given situation. This attempt is carried out by the so-called left hemispheric interpreter, a specialized module that tries to make sense of unconsciously determined behaviors (Funk and Gazzaniga, 2009). Differences in opinion on moral topics may be based on the sensitivities of specific neural circuits that process various moral dimensions (Haidt, 2007).

**PREMISE 3**. The choice of target appears to be the most relevant issue. I hypothesize that one way to bring the psychopathic brain under control is to engage “moral/compassion circuits”. Several theories concerning the neural basis of human morality have been put forth (Moll et al., 2005). One which has particular appeal is Greene (2009)’s dual-process theory of moral judgement. Accordingly, both intuitive emotional responses and more controlled cognitive responses play crucial, and in some cases, mutually competitive roles. This theory associates controlled cognitive processing with utilitarian moral judgement aimed at promoting the “greater good”; in contrast, this theory associates intuitive emotional processing with deontological judgement aimed at respecting rights, duties, and obligations that may trump the greater good. Here, the DLPFC plays a major role both in utilitarian moral reasoning (BA46) and cognitive control (BA10); BA10 may be particularly engaged by high-conflict personal dilemmas. Incidentally, the DLPFC also plays a role in racial bias, and studies show that attempts to get people to not “see” race will be relatively ineffective, but interventions that seek to improve behavior regulation capabilities (DLPFC function) might be effective in at least reducing the behavioral expression of bias (Ito and Bartholow, 2009). On the other hand, non-utilitarian intuitive emotional response is processed by BA9/10 plus the posterior cingulate cortex and posterior superior temporal sulcus/temporoparietal junction (BA39).

## Hypothesis

The first attempt at non-invasive therapeutic cortical stimulation (CS) was at the hands of Giovanni Aldini, Luigi Galvani’s nephew, at the end of the 1790s, who stimulated a depressed subject with the forerunner of modern transcranial direct current stimulation (tDCS) (Canavero, 2011).

Cortical neuromodulation can be achieved both invasively and non-invasively. The first involves the positioning of one or more stimulating paddles (ca. 5 cm × 1 cm × 2 mm) below the skull bone overlying the dura mater (which obviates any risk of intracerebral hemorrhage/infection) with a minimally invasive approach (simple burr holes, no craniectomy) under neuronavigation conditions. A connecting wire is tunneled to a subcutaneously installed subclavear pacemaker (which can be recharged from an external source). Non-invasive stimulation – which can also be employed for preoperative assessment of possible cortical targets for implanted electrodes – exploits transcranial magnetic stimulation (TMS), which is more cumbersome and expensive, but more accurate, or tDCS, which is much less expensive, but coarser in its modulatory capacity. TMS is FDA-approved for the treatment of depression, and both are currently being evaluated for other psychiatric conditions. No mortality or disabling, permanent morbidity has been reported for CS (Canavero, 2009, 2011). Recently, a tDCS study showed that stimulating the right DLPFC increases compliance to social norms enforced by punishment (Ruff et al., 2013). This point is important: psychopaths may believe to act appropriately, and it is imperative to “switch” their right/wrong circuitry to a socially non-disruptive mode. This also means that CS will be applied simultaneously to psychological reconditioning: this is similar to boosting neuroplasticity via CS during rehabilitation for stroke (see Canavero, 2009).

I propose that psychopaths be submitted to experimental CS for the behavioral control of their symptoms. Initially, targets would include BA10/46 and BA39. Their value can be assessed by either surgical implantation of electrodes for continuous or intermittent stimulation or once/twice-a-day sessions of tDCS/TMS applied for 2–4 weeks daily (eventually with a portable helmet) and psychosocial assessment, for example by challenging the subjects with behavioral prompts in a controlled setting. Serial neuroimaging would help assess brain changes. Importantly, CS has been shown to be able to permanently alter pathologic circuits (Canavero, 2009, 2011) and it is thus possible that long-term modulation may be stopped after some time once the aberrant circuits have been “remodeled.” Neuroimaging (e.g., DTI, default-state fMR, and voxel-based MR morphometry) may assess such changes.

At a later stage, individualization might be achieved. Currently, closed-loop (feedback-engaged) CS is possible for epilepsy, whereby intracranial electrodes record pathologic electrographic activity and abort it via an electric current delivered by a pulse generator, which acts both as an EEG analyzer/stimulator (Canavero, 2009). If the electrographic signature of anomalous behavior can be picked out in the single subject, a similar apparatus might abort a behaviorally inappropriate “bout of aggression.” Conceivably, neurochemical monitoring of brain areas (Van Gompel et al., 2010) involved in criminal behavior could also be applied to engage on-demand CS.

The concept of equipping a criminal subject with an “electric gadget” is not so far-fetched, considering the use of tracking bracelets of criminals for police control.

Electrical stimulation might emerge as a more humane treatment of criminal psychopaths in this century and should be pursued actively despite the legal and ethical dilemmas.
